# Preservation of Leeches

**Published:** 1860-10

**Authors:** 


### Preservation of Leeches.

We call attention to the selected article under this head, on page 98 of this Journal. The apparatus of M. Vayson, there described, strikes us as an admirable arrangement for the purpose of preserving leeches. To be able to accomplish this is of immense benefit to the country practitioner. Leeching is the very best mode of local deptetion, in many cases, and without the means of preserving them, it is impossible to command them at all times when they are required.

